# Insufficient physical activity level among Sahrawi adults living in a protracted refugee setting

**DOI:** 10.1186/s12889-021-10217-w

**Published:** 2021-01-19

**Authors:** Eivind Andersen, Ida Kjellså, Victoria Telle Hjellset, Sigrun Henjum

**Affiliations:** 1 Faculty of Humanities, Sports and Educational Science, University of South-Eastern Norway, PO box 235, 3603 Horten, Kongsberg Norway; 2 Department of Nursing and Health Promotion, Faculty of Health Sciences, Oslo Metropolitan University (OsloMet), Oslo, Norway

**Keywords:** Sahrawi refugees, Physical activity, IPAQ, Public health

## Abstract

**Background:**

The Sahrawi people fled their homes in 1975 as the conflict in Western Sahara escalated and settled down near Tindouf, Algeria, where they still live. High prevalence of overweight and obesity and type 2 diabetes had been found in this protracted refugee setting. Scaling up national policy efforts to promote physical activity (PA) is critical to reducing the prevalence of noncommunicable diseases (NCDs) in the near future. One possible barrier to the inclusion of PA in NCD prevention strategies is the lack of research on PA level, which may reduce political support and policy development for PA. Thus, the aim of this study was to investigate the PA level among adults living in Sahrawi refugee camps and socioeconomic factors associated with PA.

**Methods:**

A cross-sectional survey was carried out in 2014 in five refugee camps near Tindouf, Algeria. A total of 180 women and 175 men were included. PA was measured using the international physical activity questionnaire short form (IPAQ-SF).

**Results:**

There was a large amount of variance in reported PA for both genders, ranging from 10 min of total PA per week to above 40 h. Forty-three percent of the participants had a low PA level (defined as not meeting the PA recommendations of 150 min of moderate to vigorous PA per day). The chi-square test of independence showed that males, those aged ≥ 60 years and people with higher education were more likely to be in the low PA level category. No significant relationship was found between PA level and BMI status. Most of the participants thought that engaging in PA would be wise, valuable, right and good but thought to a lesser degree that PA would be easy, comfortable and interesting.

**Conclusions:**

Almost half of the participants were categorised as insufficiently physically active, however, most of the Sahrawi refugees express a positive attitude towards PA. PA is a low-cost approach to reducing deaths and NCDs, government initiatives to increase PA levels in refugee camps are advised.

**Supplementary Information:**

The online version contains supplementary material available at 10.1186/s12889-021-10217-w.

## Background

Western Sahara is a conflicted region to which both Morocco and the Sahrawi people claim rights [1]. The armed struggle between the two parties forced thousands of Sahrawi’s to flee east over the border to Algeria in 1975. The Sahrawi refugees settled down in refugee camps in the Tindouf Province in Algeria, where they still live. The harsh desert environment makes the refugees dependent on food aid. Monthly they receive basic food rations with dry foods as well as fresh foods such as fruit and vegetables from the international community [2]. In addition, food is sold in local shops, but the selection is scarce [2]. Furthermore, a study by Morseth et al. (2017) found that only 1/3 of the refugees had adequate dietary diversity [3]. The camps differs from the majority of other refugee camps in which camp life is organised by the refugees themselves, with little outside interference [1].

Grijalva-Eternod et al. (2012) found a coexistence of undernutrition and overweight in this protracted refugee setting, with 25% of the refugee households affected by the double burden of malnutrition [4]. Furthermore, 53.7% of the women were classified as overweight or obese, and 71.4% had central obesity. The reasons for this high prevalence of overweight and obesity are not known, but suggested factors are cultural associations between a large body and wealth and beauty [5] and excessive sugar consumption [6]. In addition, urban Sahrawi women with high body mass index (BMI) values have been found to walk significantly less than those with normal BMI values, thereby reducing their energy expenditure [6]. Additionally, a high prevalence of type 2 diabetes (T2D) and prediabetes has been found among Sahrawi refugees [7], and numbers are expected to rise in the near future (Henjum et al. 2020, in press).

Physical activity (PA) has consistently been shown to be independently associated with a lower risk of a range of chronic noncommunicable diseases (NCDs) [8, 9] and has been a primary focus of public health guidelines for many years [9, 10]. PA is also a cornerstone in the prevention and management of overweight and obesity [11], which are strongly linked to the risk of T2D [12], cardiovascular diseases (CVDs) [13, 14] and overall mortality [15]. The extent of change in CVD risk factors in response to a lifestyle intervention is related to the extent of body weight loss [16], and loss of body fat is likely to be an important mediator between changes in PA and changes in risk factors for cardiometabolic diseases [17]. However, an increase in PA has been shown to change biomarkers of cardiometabolic risk without resulting in a concomitant reduction in body weight [18].

Almost three-quarters of all NCD deaths occur in low- and middle-income countries (LMICs) [19]. This reflects the epidemiologic transition from infectious to chronic diseases in LMICs [20]. In 2011, a United Nations (UN) summit put NCDs on the global agenda and called for an international response to address the burden of these diseases throughout all parts of the world, particularly LMICs [21]. Following the UN summit, the World Health Organization (WHO) developed a global monitoring framework to enable global tracking of progress in preventing and controlling NCDs [22]. Physical activity was one of the nine targets outlined in the framework and provides a strong mandate for addressing PA globally. Scaling up national policy efforts to promote PA is critical to reducing the prevalence of NCDs in LMICs and achieving the targets set by the WHO [22]. One possible barrier to the inclusion of PA in NCD prevention is the lack of research evidence on PA levels in LMICs and thus among Sahrawi refugees, which may reduce political support and policy development for PA. Thus, the aim of this study was to investigate the type and amount of PA among adults living in Sahrawi refugee camps and factors associated with PA.

## Methods

### Study design and sample

A cross-sectional survey was carried out in September and October 2014 in five refugee camps near Tindouf, Algeria. The total population in all five camps was estimated to be approximately 165,000. In the present study, the eligible population were all adults, both males and females, above 18 years of age living in one of the five refugee camps: Smara, El Aiune, Ausserd, Dakla and Boujdor. Individuals that were sick, bedridden or due to some reason unable to answer questions were excluded from the study.

The sample size was chosen based on an estimated prevalence of an inadequate PA level (i.e., not meeting the WHO recommendations of at least 150 min of moderate-intensity PA throughout the week, or at least 75 min of vigorous-intensity PA throughout the week, or an equivalent combination of moderate- and vigorous-intensity activity (MVPA) [23]) of 50% and an absolute precision of 5% for the 80% confidence interval [24] Assuming incomplete sampling from 10% of the participants, we calculated a final desired sample size of 180 men and 180 women, as determined with Open Source Epidemiologic Statistics for Public Health (OpenEpi). Due to the unequal number of inhabitants in the five camps, a probability proportional to size (PPS) method was used to select participants from each camp [3]. We assumed a 50/50 gender balance in all the camps. This resulted in an estimated sample of 51, 51, 38, 23 and 17 persons of each gender from Smara, El Aiune, Ausserd, Dakla and Boujdor, respectively [3]. During the study period, 52 participants withdrew mainly due to work obligations or personal or family illness, and two participants were excluded. Forty-nine additional participants were randomly recruited, so that the final sample consisted of 355 participants: 175 men and 180 women. Three hundred three participants reported some form of PA and are thus included in the PA analysis.

A three-staged cluster sampling was performed. The first stage was to select camps (PPS) and the second stage was to select households. They were randomly selected by tossing a pen in order to decide the direction in which the research team was to drive upon leaving the dispensaries. The team drove toward the boarder of the camp and each seventh household was selected. The third stage was to randomly select one man and one woman from each household. If more than one man or woman in the household was eligible and wished to participate, numbers were assigned to each person. A fieldworker then pointed to a piece of paper with numbers in random order and the person who matched the selected number was included. In households where men were not present, the woman was included and a man in the neighboring household was asked to participate.

### Measurements

#### Physical activity

The international physical activity questionnaire short form (IPAQ-SF) [25] was used to assess PA. Local field workers fluent in English, Spanish or Hassania (the local language) interviewed the participants in their own homes. All field workers were trained in interview technique over a 7 days period, including 2 days for conducting a pilot study. The researchers oversaw all interviews. After the interview the researcher checked the answers and asked both the interviewer and the participant some control questions to make sure that the interviewer had asked the questions in the right manner and that the participant had understood the questions. The IPAQ focuses on the amount of PA performed over the past 7-day period. The IPAQ includes questions about the time spent engaging in vigorous PA, moderate PA and walking in 10-min bouts or longer. Within these domains, participants are asked to consider all types of physical activities, including activities performed during leisure time, domestic and gardening activities, work-related activities and transport-related activities. Relevant examples of activity performed at the different intensities were mentioned in the items. The data obtained from the IPAQ were used to estimate the total amount of PA completed in a 7-day period by weighting the reported min per week in each domain by a metabolic equivalent (MET) energy expenditure estimate. The weighted MET minutes per week were then calculated by multiplying the duration (minutes), frequency (days) and MET intensity and then summing the three domains, namely, vigorous (8 METs), moderate (4 METs) and walking (3.3 METs), to produce a weighted estimate of total PA per week (min∙week^− 1^) [25]. The participants were categorised into three PA levels according to the IPAQ scoring criteria: *Low (category 1):* meets neither moderate nor high criterion. *Moderate (category 2):* meets any of the following three criteria: (a) three or more days of vigorous-intensity activity of at least 20 min∙day^− 1^, (b) five or more days of moderate-intensity activity and/or walking of at least 30 min∙day^− 1^, and (c) five or more days of any combination of walking, moderate-intensity, or vigorous-intensity activities achieving a minimum of at least 600 MET∙min∙week^− 1^. *High (category 3):* meets any one of the following two criteria: (a) vigorous-intensity activity on at least 3 days and accumulating at least 1500 MET∙min∙week^− 1^ and (b) seven or more days of any combination of walking, moderate intensity, or vigorous-intensity activities accumulating at least 3000 MET∙min∙week^− 1^. Those categorised as having moderate or high activity were classified as being sufficiently active according to the WHO PA guidelines [23]. Data cleaning and processing were carried out in accordance with the guidelines published by the IPAQ Research Committee, and the methods used to score the IPAQ are described in the IPAQ scoring protocol [26]. Additional questions (supplementary material online), including views of one’s own PA level on a 5-point scale (0=far too low, 1=slightly too low, 2=satisfying, 3=slightly too high and 4=far too high), views regarding engaging in PA on a 3-point scale (e.g. 0=silly, 1=neutral, 2=wisely), what activities they usually engaged in, what they liked to do, and organised physical activities they would like to be arranged in the camps, were included in the questionnaire.

#### Anthropometric measures

Weight was measured with the participants in light clothing using digital Tara scales produced for UNICEF (SECA 890; SECA, Hamburg, Germany). Participants’ height, without shoes, was measured to the nearest 0.1 cm using an ultrasonic metre from Soehnle Professional (Backnang, Germany) or a UNICEF portable stadiometer if the participant had a similar or taller height than the field worker performing the measurements. Waist circumference was measured by trained personnel midway between the hipbone and lowest rib using an ergonomic measuring tape from SECA (SECA 201; SECA). A waist circumference ≥ 80 cm and ≥ 94 cm, for women and men respectively, was considered high [27]. BMI was calculated as body weight divided by squared body height (kg^.^m^− 2^).

### Statistical analysis

All statistical analyses were performed using SPSS (Statistical Package for the Social Sciences for Windows, version 26, IBM, Inc., Chicago, IL, USA). Continuous variables are presented as medians and interquartile ranges (IQRs) since they were not normally distributed, and categorical variable are presented as percentages. Differences between groups were assessed by the Mann-Whitney U test for continuous variables and Pearson’s chi-squared test for categorical variables. Associations between sociodemographic (gender (man/woman), age (18–59/60–90), education (up to 6th grade/7 to 9th grade/10 to 12th grade/higher education), marital status (married/not married), work status (paid work/not paid work) and anthropometric factors (BMI (underweight/normal weight/overweight/obese)) and low PA level (category 1 in IPAQ) were assessed with multivariable logistic regression, with low PA level as the dependent variable. All significance tests were two-sided.

### Ethics

Ethics approval for the survey was given by the Regional Committees for Medical and Health Research Ethics in Norway (ref. 2014/1155) and by the Saharawi Ministry of Public Health.

Informed consent was obtained from all participants both orally and in writing. It was emphasised that refusal to participate or withdrawal from the survey would not have any negative consequences for the participants. The study was conducted according to the guidelines provided in the Declaration of Helsinki.

## Results

The distribution of the 350 included participants were: Boujdor *n*=35, Ausserd *n*=74, El Aiune *n*=104, Dakhla *n*=42 and Smara *n*=100. Table 1 displays the relevant sociodemographic and anthropometric information for both women and men. Most of the refugees were born in the camp or had been living there since the refugee camps were set up in 1975/76. Those who had not been born in the refugee camp had been living there for a median (IQR) of 36.0 (15) years and 31.0 (24) years for women and men, respectively. A considerably larger number of women were overweight or obese than men (64.5 and 25.1%, respectively). While more than half of the men had paid work (army, construction worker, taxi driver, salesperson), very few women had paid work (kindergarten, nurse, teacher, nanny, secretary, cleaning). A total of 85.6% of the women and 89.1% of the men had some income (mostly from other members of the household). There were no differences between genders with regard to education level or marital status.

**Table 1:** Sociodemographic and anthropometric characteristics for women and men living in Sahrawi refugee camps in Algeria (*n*=355). Data are median (IQR) or percent

Characteristic	Women (***n***=180)	Men (***n***=175)	***P*** value
Age (years)	40.0 (23)	38.0 (32)	0.59
Height (cm)	157.0 (8.4)	168.5 (10.0)	<0.01
Weight (kg)	66.7 (19.5)	60.7 (18.8)	<0.01
BMI (kg^.^m^−2^)	26.9 (7.3)	21.6 (6.1)	<0.01
BMI categories			
- < 18.5 kg^.^m^−2^ (%)	2.2	18.9	
- 18.5 to 24.9 kg^.^m^−2^ (%)	33.3	56.0	
- 25.0 to 29.9 kg^.^m^−2^ (%)	31.7	20.0	
- ≥ 30 kg^.^m^−2^ (%)	32.8	5.1	<0.01
Waist circumference (cm)	91.0 (18.6)	79.7 (20.3)	<0.01
- Women: ≥ 80 cm, Men: ≥ 94 cm (%)	75.0	20.6	
Education level (%)			
- None	32.2	27.4	
- Up to 6th grade	27.2	18.9	
- 7th to 9th grade	25.0	28.6	
- 10th to 12th grade	12.2	19.4	
- Higher education	3.3	5.7	0.09
Married (%)	59.4	55.4	0.44
Paid work (%)	13.3	54.3	<0.01
Number of children	3.0 (4)	1.0 (5)	0.01
People living in household	6.0 (4)	7.0 (4)	0.03
Born in camp or lived there since the beginning in 1975/76 (%)	92.8	80.0	0.001

Abbreviations: *IQR* Interquartile range; *CI* Confidence interval; *BMI* Body mass index

Of the total sample of 355 participants, 303 reported some form of PA and are thus included in the PA analysis below. Compared to those who reported some form of PA, those who did not report any PA (*n*=52) where older (median (IQR) 37.0 (26) years vs. 56.5 (34) years, U=4741; P<0.01) had a larger waist circumference (median (IQR) 85 (20) cm vs. 92 (21) cm, U=6434; *P*=0.04) and were less educated (*P*=0.01). Women reported significantly less walking per week, more moderate PA, and less vigorous PA than men did. There were no differences in total PA levels between genders (Table 2). There was a large variance in reported PA for both genders, ranging from 10 min of total activity time per week to above 40 h, as displayed by the large IQR in Table 2.

**Table 2:** Self-reported physical activity level and energy expenditure for women and men living in Sahrawi refugee camps in Algeria (*n*=303). Data are median (IQR)

Physical activity variable	Women (***n***=155)	Men (***n***=148)	***P***-value
Walking (min∙week^−1^)	45 (105)	70 (120)	0.01
Walking (MET∙min∙week^−1^)	148 (347)	231 (396)	0.01
Moderate PA (min∙week^−1^)	105 (210)	0 (60)	<0.01
Moderate PA (MET∙min∙week^−1^)	420 (840)	0 (240)	<0.01
Vigorous (min∙week^−1^)	0 (60)	15 (140)	0.01
Vigorous PA (MET∙min∙week^−1^)	0 (480)	120 (1120)	0.01
Total activity time (min∙week^−1^)^a^	270 (433)	180 (400)	0.18
Total PA (MET∙min∙week^−1^)^a^	1129 (2013)	773 (2389)	0.39

Abbreviations: *IQR* Interquartile range; *MET* Metabolic equivalent; *PA* Physical activity

^a^Walking + moderate PA + vigorous PA [26]

Table 3 shows the associations between sociodemographic and anthropometric factors and low PA, where low PA is defined as having less than half an hour of at least moderate intensity PA on most days. Females, those aged below 60 years and those with a lower education level had a significantly lower odds ratio of having a low PA level than males, those aged 60 years or above and those with a higher education level (Table 3). There were no significant associations of BMI status, marital status or whether the participant had paid work with PA level.

**Table 3:** Association between sociodemographic and anthropometric factors and low^a^ physical activity level among people living in Sahrawi refugee camps in Algeria. Data are odds ratios (with 95% CIs) derived from multivariable logistic regression (*n*=303)

Characteristic	Odds ratio	95% CI	***P***-value
Female vs. male	0.46	0.25 to 0.86	0.01
Age 19–59 vs. ≥ 60 years	0.37	0.16 to 0.86	0.02
BMI categories vs. obese			
- Underweight	1.41	0.50 to 3.96	0.5
- Normal weight	0.90	0.42 to 1.92	0.7
- Overweight	1.55	0.71 to 3.38	0.2
Education categories vs. higher education			
- Up to 6th grade	0.29	0.14 to 0.58	< 0.01
- 7th to 9th grade	0.36	0.19 to 0.68	< 0.01
- 10th to 12th grade	0.26	0.13 to 0.52	< 0.01
Married vs. not married	0.71	0.42 to 1.21	0.22
Paid work vs. no paid work	1.21	0.68 to 2.17	0.5

Abbreviations: *BMI* Body mass index

The model is adjusted for all variables in the respective columns

^a^Low PA level was defined as those having less than half an hour of at least moderate intensity PA on most days (i.e., category 1, or low) according to the IPAQ scoring protocol [26]

**Fig. 1:**
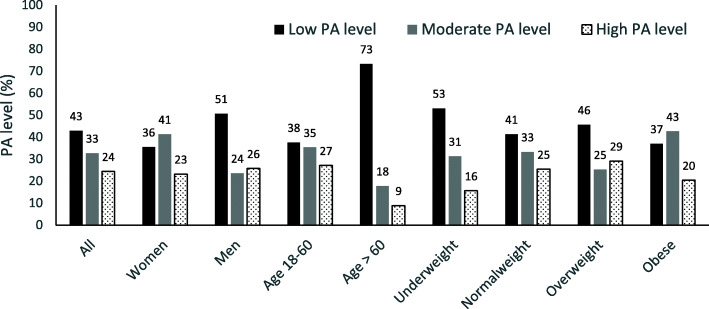
Distribution of physical activity level by gender, age and weight status (*n*=300)

Forty-three percent of the participants had a low PA level (defined as not meeting the PA recommendations of 150 min of MVPA per week) (Fig. 1). The chi-square test of independence showed that males were more likely to be in the low PA level category and less likely to be in the moderate PA level category than females (X^2^ (2) = 11.4, P < 0.01) and that those aged 60 or above were significantly more likely to have a low PA level and less likely to have a high PA level than participants under the age of 60 years (X^2^ (2) = 20.2, P < 0.01) (Fig. 1). No significant relationship was found between PA level and BMI status.

The subjective assessment of one’s own PA level showed that 27.8% of the women believed their PA level was slightly or far too low, 66.1% states that they were satisfied with their PA level and 6.1% believed that it was slightly or far too high. For the men, 23.4% reported that their PA level was slightly or far too low, 62.3% that it was satisfactory and 14.3% that it was slightly or far too high. Most of the participants thought that engaging in PA would be wise, valuable, right and good but to a lesser degree that it would be easy, comfortable and interesting (Table 4). There were no significant differences in views regarding engaging in PA between genders.

**Table 4:** The question asked, “Think about yourself being physically active the next 7 days. Is your opinion about this physical activity that it is … ” (Data are percent, *n*=302)

Women (***n***=180)			Men (***n***=175)		
Silly	Neutral	Wisely	Silly	Neutral	Wisely
1.7	8.9	88.3	4.0	8.0	87.9
Harmful	Neutral	Valuable	Harmful	Neutral	Valuable
3.9	5.6	90.6	4.0	5.7	89.7
Not necessary	Neutral	Necessary	Not necessary	Neutral	Necessary
4.4	16.7	78.3	8.0	13.8	77.6
Wrong	Neutral	Right	Wrong	Neutral	Right
0.6	6.1	92.8	1.7	8.6	89.7
Difficult	Neutral	Easy	Difficult	Neutral	Easy
15.0	25.6	59.4	15.5	21.8	62.6
Uncomfortable	Neutral	Comfortable	Uncomfortable	Neutral	Comfortable
3.9	20.6	75.6	8.0	19.0	73.0
Bad	Neutral	Good	Bad	Neutral	Good
2.8	9.4	87.8	1.1	10.9	87.9
Boring	Neutral	Interesting	Boring	Neutral	Interesting
8.3	23.9	67.8	5.7	20.7	73.6

When asked what PA they preferred (*n*=350), the most reported activities were walking (*n*=209), football (*n*=57), dancing (*n*=22) and chore-related activities such as cleaning (*n*=35) and working (*n*=17). Almost all of the participants had one or more suggestions regarding activities or sport facilities they would like to be arranged or built to help them become more physically active. The most common suggestions were sports hall (25%), football groups (24%), swimming pool (15%) and walking/running groups (10%).

## Discussion

To our knowledge, this is the first study to measure PA level in a refugee setting. Almost half of the refugees did not reach the recommended PA level. Males, those aged above 60 years and refugees with higher education had a significantly higher odds ratio of having a low PA level. No significant association between BMI and PA level was found. There was a large variance in reported PA for both genders, ranging from 10 min of total activity time per week to above 40 h. Women reported significantly less walking min per week, more moderate PA, and less vigorous PA than men. Most of the participants thought that engaging in PA would be wise, valuable, right and good but to a lesser degree that it would be easy, comfortable and interesting. Most of the Sahrawi refugees included in this study were born in the camp or had been living there since the camps were established in 1975/76. Approximately one-third of the participants had no education, and only a few percent had higher education. There was a relatively large difference between genders with regard to both BMI and waist circumference, where 64.5% of the women and 25.1% of the men were classified as overweight or obese according to their BMI, and 75% of the women and 20.6% of the men had a waist circumference above the recommended level [27]. One possible explanation for the higher BMI and waist circumference in women compared to men might be that women to a larger degree prefer a larger body size [28], because this is associated with richness, health, strength, and fertility [29].

Forty-three percent of the participants were categorised as insufficiently physically active, which is a higher prevalence than what was found in a study including 146 countries (93% of the world’s population) where 23% were found to be insufficiently physically active [30]. An even lower prevalence of 15% was found among people living in low-income countries [30]. A significantly lower prevalence of low PA in low-income countries was also found in the PURE study (21%) [14] and a World Health Survey-based study (28%) [31]. There might be several reasons for the low PA level among the Sahrawi refugees. The most striking factor may be the harsh desert environment, with extremely hot summers and windy and cold winters, which makes it difficult to be physically active [32]. Another likely significant factor is that more than half the Sahrawi population is considered particularly vulnerable by the UN refugee agency (UNHCR) due to their heavy reliance on humanitarian assistance to access food, water, and other necessities [33], which would most likely make PA a non-prioritised behaviour to engage in. Other factors explaining the low PA level in our sample might be the high unemployment rate, low education level, and the relatively high age [34] and the few recreational activities offered in the camps. Although the participants with a higher education were significantly less physically active than those with less education, these people were mostly employed in sedentary jobs. In contrast to what was found in a study of 122 countries [35], women were less likely to have a low PA level than men. However, our finding is in line with a multinational study including 46 LMICs with over 200,000 participants that also found women to be more physically active than men [36]. A possible explanation might be that the women are responsible for the daily household chores and errands and thus reach the recommended levels of PA through these activities. At the same time, men are either unemployed and sit at home with little to do, or if employed, work in jobs that are mostly sedentary. An unexpected finding is that there was no association between PA level and BMI status, which is in contrast to the vast majority of research findings (e.g., [37–39]). Seeing that almost all of the obese participants were women, many of these participants are believed to walk for at least 30 min on five or more days of the week (PA recommendations using IPAQ) because of their household duties.

CVD is the leading cause of death worldwide [40] and a major economic global burden [41]. While CVD mortality has been reduced in high-income countries, the prevalence of CVD and CVD mortality has increased in low-income countries [42]. As shown in many studies from high-income countries [43], Lear et al. (2017) found in a study on almost 30,000 people from low-income countries that meeting the WHO PA recommendations was associated with a 22% lower risk for all-cause mortality and a 20% lower risk of major CVDs than the respective risks associated with not meeting the PA recommendations [14]. The high number of participants in the current study not meeting the PA recommendations is thus a cause of concern. As participating in PA is inexpensive, PA is a low-cost approach to reducing deaths and CVD that is applicable with a large potential effect [14]. Furthermore, the results from Lear et al. (2017) provide robust evidence to support public health interventions to increase all forms of PA in countries with different socioeconomic circumstances [14].

Most of the Sahrawi refugees in our sample report that engaging in PA would be wise, valuable, right and good. In addition, considering the low PA level in this population and the high overweight and obesity rates (especially among women) and thus the increased risk of NCDs putting more pressure on the health system, addressing PA seems appropriate. A systematic review of NCD policies in LMICs concluded that most countries are poorly prepared to tackle the NCD epidemic [44]. Only a few LMICs have developed national policies aimed at reducing NCDs, and even among those with national NCD policies, PA is rarely included [44]. Scaling up national policy efforts to promote PA is critical to reducing the prevalence of NCD in LMICs and achieving the targets set out in the Global Monitoring Framework [45]. There seems to be, however, important barriers to policy implementation that must be overcome before progress in increasing PA can be expected (i.e., PA surveillance, research, resources) [30]. A Lancet review found a small but increasing number of intervention studies from LMICs, and these studies provide initial evidence that community-based interventions can be effective [30]. The authors further conclude that greater implementation of evidence-based interventions could help control NCDs in LMICs [30].

### Strengths and limitations

The main strength of this study is the relatively large, randomly selected sample including both women and men and a high response rate. Our study also has some limitations that need to be addressed. First, the extent to which causality can be inferred is lower than that associated with a longitudinal study due to the cross-sectional design used in this study. Second, although the IPAQ-SF is recommended and widely used, validation studies show only a small correlation with objective measures [46]. Most of the studies included in the systematic review found that the IPAQ-SF overestimated PA levels by 36 to 173% [46]. In a large population-based validation study in Norway, an underestimation of moderate-intensity PA and overestimation of vigorous intensity PA were found [47]. Furthermore, the difference between the IPAQ-SF and accelerometer-measured MVPA increased with higher activity and intensity levels and was further affected by male gender, older age and low education level [47]. Although the IPAQ-SF has shown reasonable reliability and can be used to compare PA levels between different populations [25] it has not been validated on this specific population. It is also a weakness of the study that, because of the language barrier, the researcher could not conduct the interviews themselves. However, the researchers oversaw all interviews and control questions were asked to both the interviewers and the participants. The interviewers were also trained for 7 7 days, and which included a pilot study.

## Conclusions

Almost half of the Sahrawi refugees do not reach the recommended PA level. Males, those aged above 60 years and people with higher education were more likely to be in the low PA level category. Most of the participants expressed a positive attitude towards PA and seeing that PA is a low-cost approach to reducing deaths and CVD, government initiatives to increase PA levels in refugee camps are advised.

## Supplementary Information

**Additional file 1.**

## Data Availability

Data can be accessed upon request to Eivind.andersen@usn.no.

## Abbreviations

PA: Physical activity
NCDs: Noncommunicable diseases
IPAQ-SF: International physical activity questionnaire short form
BMI: Body mass index
T2D: Type 2 diabetes
CVDs: Cardiovascular diseases
LMICs: Low- and middle-income countries
UN: United Nations
WHO: World health Organization
MVPA: Moderate- and vigorous-intensity activity
MET: Metabolic equivalent
IQR: Interquartile range
CI: Confidence interval
UNHCR: UN refugee agency
