# Supplementary material for: Insufficient physical activity level among Sahrawi adults living in a protracted refugee setting
Source: BMC Public Health. 2021 Jan 19;21:166. doi: 10.1186/s12889-021-10217-w (PMC7816400; doi:10.1186/s12889-021-10217-w)
Supplement: Supplementary file 1 — Additional file 1. [file 12889_2021_10217_MOESM1_ESM.docx]

**Supplementary file**

1. Do you believe that your level of physical activity is;
   far too low, slightly too low, satisfying, slightly too high, or far too high?
   0= far too low, 1=slightly too low, 2=satisfying, 3=slightly too high, 4=far too high
2. Think about yourself being physical active the next 7 days. Is your opinion about this physical activity that it is:
   1. Silly or wisely?

0=Silly, 1=neutral, 2=wisely

- 1. Harmful or valuable?

0=Harmful, 1=neutral, 2=valuable

- 1. Not necessary or necessary?

0=Not necessary, 1=neutral, 2=necessary

- 1. Wrong or right?

0=Wrong, 1=neutral, 2=right

- 1. Difficult or easy?

0=Difficult, 1=neutral, 2=easy

- 1. Uncomfortable or comfortable?

0=Uncomfortable, 1=neutral, 2=comfortable

- 1. Bad or good?

0=Bad, 1=neutral, 2=good

- 1. Boring or interesting?

0=Boring, 1=neutral, 2=interesting

1. Which of the following activities do you usually engage in once or more during a week? (Read the alternatives and mark 0=no or 1=yes, multiple answers are possible)
   0=No, 1=Yes
   1. Walking
   2. Running
   3. Cleaning
   4. Farming/gardening
   5. Working with animals
   6. Digging
   7. Lifting
   8. Playing football
   9. Dancing
   10. Other? Please specify
2. What type of physical activity do you like to do?
   ……………………………………………………………………………………………..
3. Is there something that could have been different to keep you more physical active? (e.g. arranged dancing groups, football matches, having sport halls, swimming pool etc.)…………………………………………………………………………………………
4. Can you suggest something that may help others to be in physical activity?

……………………………………………………………….
